# Book Review: The Courage to Suffer: A New Clinical Framework for Life's Greatest Crises

**DOI:** 10.3389/fpsyg.2020.617236

**Published:** 2020-12-03

**Authors:** Claude-Hélène Mayer

**Affiliations:** ^1^Department of Industrial Psychology and People Management, University of Johannesburg, Johannesburg, South Africa; ^2^Institut für therapeutische Kommunikation und Sprachgebrauch, Europa Universität Viadrina, Frankfurt, Germany

**Keywords:** suffering, crisis, growth, pain, therapy, clinical framework

## Introducing the Book and the Authors

Daryl R. Van Tongeren and Sara A. Showalter Van Tongeren's book “The Courage to Suffer” seems to have been published at just the right moment, at the beginning of 2020, the year in which humankind faces one of the biggest global health-related crises in history. The book is dedicated to the authors' clients and students who have shown how working with suffering and the deep experience of pain and loss can lead to flourishing and well-being.

The authors, who combine their well-established theoretical and practical knowledge and parts of their personal and professional life experiences into a “new clinical framework,” have divided the book into eight chapters. They conclude with an epilogue which presents the reality of the pain, suffering, and loss that humans experience, together for the potential for making meaning, while being aware of the existential realities of life, thereby creating flourishing.

The book differs from other books published in the field of existential psychology in so far that the authors have extraordinarily combined their own life experiences with their theoretical background and their practical clinical work. The reader can therefore explore the topic of how to overcome suffering and pain on personal, theoretical, and practical levels. The book is therefore a highly recommendable contribution to the field.

## Exploring the Book's Content

The book is a homage to the spirit of Viktor Frankl, the Austrian neurologist, psychiatrist, and holocaust survivor, who spent his life deciphering ways to transform suffering and create meaning and flourishing. The book very much reflects the perspective of Wong's (2020) Positive Psychology (PP2.0), in the way that it emphasizes that mental health and well-being can only sustainably be reached when individuals work through the dark side of suffering toward reaching the light. Although the authors do not mention Wong's theory, it might be seen as a contribution toward their work.

## The Five Chapters of Darkness, Suffering, and Pain

The first five chapters of the book deal with *An Existentialist Positive Psychology Framework* (Chapter 1), *The Existential Themes of Suffering* (Chapter 2), *The Sunset: The String of Suffering* (Chapter 3), *Dusk: Into the Darkness* (Chapter 4), and *Midnight: The Deconstruction Process* (Chapter 5). Chapters 6 to 8, however, deal with the question of how to reach the richness of life and the light while experiencing pain.

The authors begin on a very personal note, describing the death of the brother of one of the authors, his threat of a similar fate due to genetic circumstances, the death of a parent, the advice not to have children because of the author's condition and finally the diagnosis of infertility.

Based on the authors' own experiences of loss and suffering and their professional experiences, they develop a clinical framework which proposes to find a new way of suffering, leading to flourishing and self-development. The framework is based on Frankl's (1959) idea that a person needs “courage to suffer” and that suffering—as in many psycho-therapeutic approaches—does not need to be fixed, but rather approached in different ways: “There is no fixing of death, infertility, loss of a dream, or permanent shift of one's identity” (Van Tongeren and Showalter Van Tongeren, 2020, p. 5). Instead, they suggest that “suffering is an inherent part of life that must be engaged” (Van Tongeren and Showalter Van Tongeren, 2020, p. 5).

By developing their framework, the authors walk the tightrope of presenting their own life stories and their work with their clients, which makes the book an authentic approach to dealing with existential phenomena and their inherent potential to cause suffering. The authors further support their views and experienced and practical approaches with scientific findings and research results.

The authors' framework is an existential positive psychology perspective in which a person's core values, virtues, and relationships are identified and then, based on these core aspects, flourishing is promoted while living through the pain, thereby overcoming the inherent meaninglessness in life, human's isolation in the world and the anxiety of the certainty of death. Drawing on bestselling authors such as Viktor Frankl and Irving Yalom, the authors point out the importance of both positive psychology and existential concerns, in the midst of which “meaning can be cultivated” (Van Tongeren and Showalter Van Tongeren, 2020, p. 7). Accordingly, this book is published at a time when humankind is experiencing strong waves of uncertainty, new evolving questions of meaning in life through the Covid-19 pandemic, the recovering of virtues and the urge to seek new ways of dealing with suffering, pain and death.

The first chapter not only presents the clear viewpoint of the authors and the anchoring of their framework approach, but also grounds it in the philosophies of the giants of existentialism such as Sartre, clarifying definitions of basic concepts such as suffering and meaning. The authors present case examples of their clients, ask the reader questions to stir reflections and offer answers from their own scientific research framework. Most helpful for the understanding of suffering is the way in which their phase orientation is framed, through Sunset, Dusk, Midnight, Dawn, and Daylight (Van Tongeren and Showalter Van Tongeren, 2020, p. 7) which can either be used for own, personal understanding of suffering or for a therapist to work through with clients.

In the second chapter, the authors present existential themes of suffering, such as groundlessness, isolation, identity, and death, while explaining in depth the symptoms and expressions of these themes and their importance in life. The case examples and recommendations of the authors on how to deal with existential suffering and crisis are of particular interest.

In the following chapters (Sunset, Dusk, and Midnight), the authors guide the reader through the phases of suffering and the therapeutic approaches which therapists can use to assess and stabilize their clients (Chapter 3 Sunset), to help accept the suffering and pain (Chapter 4 Dusk), and to active questioning (Chapter 5 Midnight).

Further, each chapter presents research on how people deal with the different phases of suffering they go through. Understanding the worldviews of the clients is of major importance (Van Tongeren and Showalter Van Tongeren, 2020, p. 42). Applications are presented which range from meaning assessments to engaging in new ways of thinking and behaving, being present to stabilize the client, validating the experiences, to mindfulness practice and cost-benefit analysis.

In Chapter 4, the authors explain the discrepancy-distress dilemma (Park, 2010) which is an eye-opener for understanding the experience of distress and its origins. Active questioning is presented as the way to question one's own beliefs, which might need to be deconstructed to work through pain and suffering—which in itself may bring on even more pain. At the end of Chapter 4 comes radical acceptance and how to approach it.

The authors even provide insights into their own therapeutic practice and dialogues with clients, as well as relaxation and breathing techniques, mindfulness exercises, intellectual exploration, self-care, and groundedness. The dialogues between client and therapist are pointed, and lead the readers toward their own reflections on life and death, existential core topics, and pain-points of their own lives through the lens of the other, while providing insights and ways to deal with these experiences.

## The Three Chapters of Lightful Awakening

Chapter 6, Dawn: The Reconstruction Process, is the turning point of the book in that it suggests how this phase of suffering and pain might be experienced as being empowering, as a starting point to see more light. The dawn phase brings focus to the autonomy of the individual. The chapter guides the therapist how to lead the client to rebuild beliefs and narrate new stories in the context of the experienced suffering. Again, the authors place their ideas into the context of contemporary research, and return to the case studies they provided in Chapter 1. One highlight of the chapter is the idea of “weakening the myth of greater meaning” (Baumeister, 1991) on which the new narratives of individuals can be built, placing suffering in the context of the bigger picture of life and realities. Thereby, the therapist can draw on modeling openness, curiosity, and grace, while allowing previous beliefs to exist. The authors again use the technique of body work to install these new values through, for example, open posture practice, motivational interviewing and new ways of using one's own language. All of this can bring forward new identity formations which can be fostered through techniques such as writing stories and meditations or even reflective meaning-making.

In Chapter 7, Daylight: Living Authentically, the authors explore how to come closer to living an authentic life. They take a deeper look into how people change, and return to their case studies to explore the turning points in authentic living and meaning-making on a new level. The core messages of the chapter—that each person's path is unique (Van Tongeren and Showalter Van Tongeren, 2020, p. 115) and that a key to living coherently is “overcoming avoidance”—are presented in a lively way. These lead to the crucial concept of existential resilience which sees life experiences as “truths and not as threats” (Van Tongeren and Showalter Van Tongeren, 2020, p. 121). In application, the authors again suggest very effective ways of dealing with the new realities, such as creating motivational checklists, guided imagery, and symbol work, as well as the exploration of compassion. The book is very rich in presenting different methods of application based on individualized case examples, so that the reader can see which method can be used in which case. The book thereby provides the reader with great opportunities to deeply understand different, individualized approaches across the transformative levels how to overcome the pain and the suffering to finally develop and grow.

## Chapter 8—A Flourishing Life

The final chapter, A Flourishing Life, offers an approach to finding meaning and existential resilience by using the head (religion), heart (relationships), and hand (cultivating virtues). Being a psychologist and therapist myself, I found this chapter being a valuable summary of the entire book, including explanations, reflections and practical, down-to-earth insights on how to steer the process with self-regulation and courage in self and clients. The authors refer to huge concepts—humility, forgiveness, gratitude, patience, and hope—and explain the impact these have in creating a flourishing and meaningful life. In doing so, the authors use simple, accessible language which is extremely effective.

## Conclusions

In the way Frankl (1959) has referred to resolving challenging situations, pain, fear and anxiety, and desperation, this book by Van Tongeren and Showalter Van Tongeren presents new and practical methods to move from suffering to flourishing. The book fully endorses Frankl's famous statement “*When we are no longer able to change a situation, we are challenged to change ourselves*.” It thereby combines uniquely existential and positive psychology approaches, while drawing on research and practical experiences, as well as established therapeutic practice.

The authors describe successfully how the inner turn from suffering toward self-development can look—for themselves, for their clients, and for therapists. Their approach is based on four pillars: their personal experience, their professional mindset, their experiences with their clients, and their dive into science and research. It provides a holistic approach to dealing with existential human suffering and its transformation toward self-development and flourishing, based on the central concept of meaning-making which is anchored in a balanced and well-described research and practice.

This book manages to reach into the depths and complexities of a person's existence and philosophy of life. while using simple sentences and “easy to read and understand content.” This approach enables any person who would like to deal with their own, or their clients' pain and suffering to do so. By describing different individuals and their cases, the authors provide great insight into their work and into how they approach the diversity of their clients' identities, emotions, challenges, suffering, and pain.

This volume is a treasure chest for individuals, clients, and therapists, and for anyone who fears addressing their own pain and suffering. The authors present a gentle approach to deal with core issues in every person's life and give sensitive guidance. The content of the book is authentic—it is impressively authentic; the reader can recognize that the authors know what they are talking about, the suffering, the pain, and the resurrection toward increased flourishing and meaning. But not only that: they also present the reader with a great, practical knowledge of existential interventions—based on various cases which are well chosen—, and explain how to apply them and make them usable in therapy or for the self.

I strongly recommend this book, as an important cornerstone of theory and practice in existential positive psychology wave two (PP2.0), particularly in times such as these when humanity is experiencing a huge existential crisis in the search for new realities and new meaning.

## Author Contributions

C-HM wrote the book review.

## Conflict of Interest

The author declares that the research was conducted in the absence of any commercial or financial relationships that could be construed as a potential conflict of interest.
